# Megatrends und Strukturwandel — welche Regionen werden profitieren?

**DOI:** 10.1007/s10273-022-3194-4

**Published:** 2022-05-20

**Authors:** Mirko Kruse, Dörte Nitt-Drießelmann, Jan Wedemeier

**Affiliations:** 1 Hamburgischen WeltWirtschaftsInstitut gemeinnützige GmbH (HWWI), Fahrenheitstr. 1, 28359 Bremen, Deutschland; 2 HWWI, Oberhafenstraße 1, 20097 Hamburg, Deutschland; 3 HWWI, Fahrenheitstr. 1, 28359 Bremen, Deutschland

**Keywords:** O11, R10, R11

## Abstract

Gesellschaftliche und wirtschaftliche Dynamiken führen zu einem stetigen Wandel von Wirtschaftsstrukturen. Abhängig von der regionalen Wirtschaftsstruktur verlaufen Anpassungsprozesse einfacher oder schwerer. Die transformatorischen Veränderungen wirken sich dabei positiv oder negativ auf die wirtschaftliche Stärke einer Region aus. Der Beitrag zeichnet auf, wie sich regionale Disparitäten aktuell darstellen und welche Regionen von sich abzeichnenden Megatrends profitieren können bzw. in welchen der Anpassungsdruck zunehmen wird. Damit der Wandel gelingt, sind unterstützende politische Rahmenbedingungen und Vorleistungen der öffentlichen Hand unabdingbar.

## Abstract (en)

Structural change and transformation is not a temporary phenomenon. The regional economic structure decides whether or not structural change has a positive effect on a region. In Germany, the process of structural change encounters heterogeneous regions that differ in terms of e.g. spatial structure, demography or economic strength. Moreover, urban areas can adapt particularly well to current trends, while rural and peripheral areas threaten to fall behind. The article presents the findings of a model that assesses how well regional economic structures in Germany are adapted to upcoming megatrends as well as market and technology trends. The model assesses both future development potential and past development. It concludes that changes should not be halted by structure conserving policies but should rather be taken up proactively in order to shape change. This requires an innovation policy that is increasingly aimed at cooperation and networking.

Die Wirtschaft befindet sich im Umbruch. Dies ist keine aktuelle Besonderheit, da Strukturwandel als stetiger Prozess zu verstehen ist. Neu hingegen sind Geschwindigkeit und Umfang dieser Veränderungen, die weltwirtschaftliche, demografische und technologische Verschiebungen umfassen. Vereinzelt wird die derzeitige Gemengelage struktureller Verschiebungen gar mit „Zeitenwende" überschrieben (Vöpel, 2020). Aktuell besonders spürbar werden die Auswirkungen der Wandlungsprozesse im Zuge der Coronapandemie. Hier trifft eine krisenhafte Entwicklung aufgrund des pandemiebedingten Ausnahmezustands auf einen bereits laufenden Wandlungsprozess (Feld et al., 2021).

Die deutsche Volkswirtschaft entwickelt sich seit Jahren hin zu einer Dienstleistungswirtschaft, in der die Beschäftigung in wissensintensiven Dienstleistungsbranchen zunimmt (Hüther et al., 2019). Gleichzeitig geraten ganze Industriezweige im Zuge geopolitischer Verschiebungen und ambitionierter werdender Klimaziele unter Druck. Megatrends wie demografischer Wandel oder Digitalisierung kommen hinzu (Ehlert et al., 2019; Sachverständigenrat, 2018). Die Verschiebungen besitzen nicht nur eine branchenbezogene, sondern auch eine ausgeprägte regionale Dimension. Infolge veränderter Wertschöpfungsketten und Branchenstrukturen werden wirtschaftliche Aktivitäten und Wohlstand zwischen Regionen neu verteilt (Vöpel und Wolf, 2017).

Die deutschen Regionen sind wirtschaftsstrukturell unterschiedlich geprägt. So sind z. B. die Schwerindustrie im Ruhrgebiet, die Landwirtschaft in Niedersachsen oder der Handel und die Schifffahrt in Bremen und Hamburg von besonderer Bedeutung (Hüther et al., 2019). Da verschiedene Sektoren unterschiedlich stark von Veränderungen betroffen sein werden, werden sich auch die regionalen Auswirkungen des Strukturwandels unterscheiden.

## Megatrends und deren Auswirkungen

Megatrends stoßen Anpassungsprozesse an und werden die wirtschaftlichen Entwicklungen in Deutschland und Europa in den kommenden Jahrzehnten prägen. Ausgehend von den derzeitigen Wirtschaftsstrukturen in den 401 deutschen Landkreisen und kreisfreien Städten (Stand 2021) widmet sich der Beitrag der Frage, wie aktuelle Megatrends die künftige Beschäftigung in einzelnen Regionen beeinflussen könnten. Werden bestehende wirtschaftliche Disparitäten verstärkt oder vermindert? Eröffnen sich im Zuge der Entwicklungen Chancen für bisher abgehängte Regionen?

* Der Artikel basiert auf Kruse, M. und J. Wedemeier (2021), Strukturwandel in Regionen und dessen Bedeutung für Norddeutschland, HWWI Policy Paper 134, Hamburg.

**Mirko Kruse** ist Junior Researcher am Hamburgischen WeltWirtschaftsInstitut (HWWI).

**Dörte Nitt-Drießelmann** ist dort Senior Researcher.

**Dr. Jan Wedemeier** ist dort Senior Researcher.

Tabelle 1
**Ausgewählte Megatrends und daraus abgeleitete Markt- und Technologietrends in Europa**

| Megatrend | Markt- und Technologietrends |
|---|---|
| 1. Demografischer Wandel | 1. Betreutes Wohnen, 2. Abnehmende Bevölkerungszahlen |
| 2. Nachhaltigkeit | 3. Bioökonomie, 4. Günstige erneuerbare Energie, 5. $CO_2$-arme Wirtschaft, 6. Materielle Ressourceneffizienz |
| 3. Digitalisierung | 7. Allgegenwärtige Expertensysteme, 8. Virtuelle emotionale Intelligenz, 9. Sensortechnologie, 10. Quantentechnologie, 11. Internationale Konfrontation, 12. Raumfahrt, 13. Digitale Arbeitsanwendungen |
| 4. Sicherheit | 14. Anhaltender Cyberkrieg, 15. IKT-basierte Sicherheit und Verteidigung |
| 5. Gesundheit | 16. Bekämpfung übertragbarer Krankheiten, 17. Austausch menschlicher Organe, 18. Präzisionsmedizin, 19. Lehren aus COVID-19, 20. Virtuelle Patienten, 21. Ganzgenomsequenzierung |
| 6. Arbeit und Produktion | 22. Nano- bis Makro-Integralfertigung, 23. Neuausrichtung des Arbeitsbegriffs, 24. Basisinfrastruktur, 25. Verlagerung von Produktionskapazitäten, 26. Hyperkonnektivität, 27. Dienstleistungswirtschaft |
| 7. Ernährung | 28. System diverseren Nahrungsangebots, 29. Neue und nachhaltige Ernährung, 30. Verlässliche Nahrungsmittelversorgung |
| 8. Wissensgesellschaft | 31. Neues Wissenssystem |

Quellen: Mesloh et al. (2021); HWWI.

Tabelle 2
**Höchste und niedrigste Sektortrendwerte über 39 Anwendungsfelder**

| Rang | Code (WZ 2008) | Wirtschaftssektor | Sektortrendwert |
|---|---|---|---|
| 1 | 72 | Forschung und Entwicklung | 27 |
| 2 | 74 | Sonstige freiberufliche, wissenschaftliche und technische Tätigkeiten | 25 |
| 2 | 82 | Erbringung von wirtschaftlichen Dienstleistungen für Unternehmen und Privatpersonen a. n. g. | 25 |
| 4 | 78 | Vermittlung und Überlassung von Arbeitskräften | 24 |
| 5 | 62 | Erbringung von Dienstleistungen der Informationstechnologie | 20 |
| 5 | 63 | Informationsdienstleistungen | 20 |
| 5 | 68 | Grundstücks- und Wohnungswesen | 20 |
| 5 | 71 | Architektur- und Ingenieurbüros; technische, physikalische und chemische Untersuchung | 20 |
| …. | | | |
| 77 | 19 | Kokerei und Mineralölverarbeitung | -4 |
| 77 | 31 | Herstellung von Möbeln | -4 |
| 77 | 49 | Landverkehr und Transport in Rohrfernleitungen | -4 |
| 80 | 6 | Gewinnung von Erdöl und Erdgas | -5 |
| 80 | 50 | Schifffahrt | -5 |
| 82 | 52 | Lagerei sowie Erbringung von sonstigen Dienstleistungen für den Verkehr | -6 |
| 82 | 53 | Post-, Kurier- und Expressdienste | -6 |

Quellen: Mesloh et al. (2021); HWWI.

Für die Analyse wurden acht bedeutsame Megatrends benannt, aus denen 31 Markt- und Technologietrends abgeleitet wurden (vgl. Tabelle 1). Diese wurden weiter auf 39 konkrete praktische Anwendungsfelder heruntergebrochen (Mesloh et al., 2021), die anschließend in einer Kreuztabelle 83 Wirtschaftsabteilungen (gemäß WZ 2008) gegenübergestellt wurden. In einem ersten Schritt wurde für jeden der 83 Sektoren und für jedes der 39 Anwendungsfelder evaluiert, ob sich das jeweilige Anwendungsfeld positiv oder negativ auf die künftige Beschäftigung innerhalb des Sektors auswirken könnte oder ob keine unmittelbare Beziehung zwischen Sektor und Anwendungsfeld zu erwarten ist. Keine Verbindung wäre z. B. bei dem Sektor Kohlebergbau und dem Anwendungsfeld Zellforschung gegeben. Erwartete positive Beschäftigungseffekte eines Anwendungsfeldes auf einen Sektor wurden mit „1“, negative mit „-1“ und neutrale Entwicklungen mit „0“ kodiert. Die Summe über alle Anwendungsfelder bestimmt den Sektortrendwert, mit der die Entwicklungsprognose des jeweiligen Sektors als Zahl messbar wird. Die Wirtschaftssektoren mit den höchsten und niedrigsten Sektortrendwerten sind in Tabelle 2 aufgelistet.

## Sektorschwerpunkte und Zukunftsfähigkeit von einzelnen Regionen

In einem zweiten Schritt wurden die Lokationsquotienten[1] für jede Region in Deutschland und für jeden Sektor auf Grundlage der sozialversicherungspflichtig Beschäftigten (gemäß dem Arbeitsortprinzip) berechnet (Bundesagentur für Arbeit, 2019). Abschließend wurden Lokationsquotienten und Sektortrendwerte multipliziert und zu einem Sektorprognosewert für die jeweilige Region zusammengeführt.

1 Der Lokationsquotient wird gebildet, indem der Anteil der Beschäftigten in einem Sektor in einer Region zum entsprechenden durchschnittlichen Anteil der Beschäftigten in diesem Sektor in allen betrachteten 401 deutschen Landkreisen und kreisfreien Städten in Bezug gesetzt wird. Ein Lokationsquotient größer (kleiner) als 1 zeigt an, dass der Anteil der Beschäftigung in einer Region im Vergleich zu allen deutschen Landkreisen und kreisfreien Städten überdurchschnittlich (unterdurchschnittlich) ausfällt.

Abbildung 1

**Bewertung regionaler Wirtschaftsstrukturen – Zukunftstrends[1]**

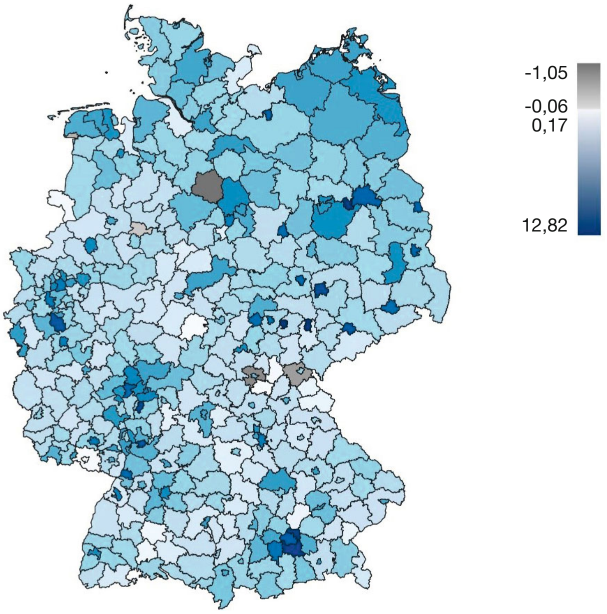

1 Mittelwerte über 83 Sektorprognosewerte auf Basis der regionalen Lokationsquotienten (Stichtag 30.6.2018) und der Sektortrendwerte.

Quellen: Bundesagentur für Arbeit (2019); Mesloh et al. (2021); HWWI.

Abbildung 2

**Bewertung regionaler Wirtschaftsstrukturen – Vergangenheitstrends[1]**

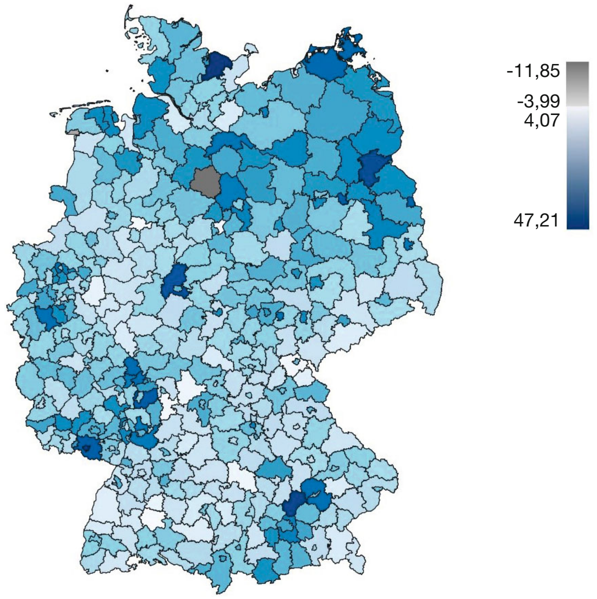

1 Mittelwerte über 83 Sektorprognosewerte auf Basis der regionalen Lokationsquotienten (Mittelwert der Jahre 2008 bis 2018) und der Sektortrendwerte.

Quellen: Bundesagentur für Arbeit (2019; 2021); Mesloh et al. (2021); HWWI.

Mit dieser Methode lässt sich die regionale Spezialisierung in einzelnen Wirtschaftssektoren sowie deren voraussichtliche Entwicklungsprognose im Kontext globaler Megatrends für jeden Landkreis und jede kreisfreie Stadt in Deutschland quantifizieren. Für die Abbildung 1 wurden für jeden Landkreis und kreisfreie Stadt aus den Sektorprognosewerten aller 83 Wirtschaftssektoren Mittelwerte gebildet. In der Farbskala sind dunkle Einfärbungen mit positiven Entwicklungsprognosen, also hohen Sektorprognosewerten assoziiert, während eine neutral weiße Einfärbung impliziert, dass sich positive und negative Trends ausgleichen oder kein besonderer Anpassungsdruck erwartet wird. Grau gefärbte Regionen stehen hingegen vor der Herausforderung, dass ihre dominierenden Wirtschaftssektoren in der Zukunft besonders stark von strukturellen Veränderungen im Zuge der Anpassungsprozesse an die acht betrachteten Megatrends betroffen sein könnten.

Um die Ergebnisse der Methodik zu verifizieren, wurde ergänzend die Beschäftigungsentwicklung der einzelnen Sektoren in Deutschland zwischen 2008 und 2018 berechnet und im selben Verfahren wie oben beschrieben auf die Regionen übertragen (vgl. Abbildung 2). Während Abbildung 1 also eine Prognose für die Zukunft darstellt, bezieht sich Abbildung 2 auf Vergangenheitsdaten. Beim Vergleich beider Abbildungen wird deutlich, dass trotz Unterschieden in der Stärke regionaler Betroffenheiten die strukturellen Muster weitgehend parallel verlaufen. Zukunftstrends wirken also mehrheitlich nicht grundsätzlich gegensätzlich, weisen jedoch teilweise eine andere Intensität als Vergangenheitstrends auf. Unterschiede zwischen den beiden Abbildungen erklären sich dadurch, dass durch die acht Megatrends Änderungen in den Entwicklungsrichtungen angestoßen werden, die sich 2008 bis 2018 noch nicht abzeichneten und daher nicht festgestellt werden konnten. Die regionalen Muster decken sich in Teilen mit anderen Untersuchungen zur Regionalentwicklung in Deutschland (BMWi, 2019; 2021; Nitt-Drießelmann und Wedemeier, 2019; Oberst et al., 2019; Oberst und Südekum, 2019; Koschatzky und Kroll, 2019).

## Strukturwandel und Anpassungsdruck in den Regionen

Auf regionaler Ebene sind in der Zukunftsbetrachtung (vgl. Abbildung 1) mehrere Auffälligkeiten zu erkennen. So sind Regionen, denen eine positive künftige Entwicklung aufgrund ihrer derzeitigen Wirtschaftsstruktur unterstellt wird, teilweise deckungsgleich mit jenen Regionen, die derzeit noch Aufholbedarf (vgl. Abbildung 2) besitzen. Die

Hansestadt Rostock (Mecklenburg-Vorpommern) und die Landkreise Landsberg am Lech, Traunstein (beide Bayern), Tübingen und Hohelohnekreis (beide Baden-Württemberg) sind Beispiele für die Regionen, in denen der Vergangenheitstrend deutlich unter dem Durchschnitt, der Zukunftstrend jedoch über dem Durchschnitt aller Landkreise und kreisfreien Städte liegt. Andererseits können auch Regionen identifiziert werden, in denen der Vergangenheitstrend deutlich über dem Durchschnitt, der Zukunftstrend jedoch weit unterdurchschnittlich ausfällt. Insbesondere der Landkreis Südwestpfalz und die Stadt Pirmasens (beide Rheinland-Pfalz), aber auch die Landkreise Freising (Bayern) und die Stadt Kassel (Hessen) könnten aufgrund der Megatrends unter Anpassungsdruck geraten. Für den Landkreis Celle und die Stadt Emden (beide Niedersachsen) ist keine Trendumkehr in Sicht. Der negative Trend der Vergangenheit setzt sich auch in der Zukunft fort. Negative Auswirkungen der Megatrends werden für die Landkreise Coburg, Hof (beide Bayern) und Herford (Nordrhein-Westfalen) prognostiziert. Hier wird erwartet, dass der in der Vergangenheit noch positive Trend ins Negative dreht, sodass erhöhter Handlungsbedarf besteht.

Ins Auge sticht, dass besonders gute künftige Entwicklungsaussichten vorwiegend in kreisfreien Städten und Großstädten verortet sind. Da die Bewertung der regionalen Zukunftsfähigkeit in unserem Modell anhand von Wirtschaftssektoren erfolgt und die besonders chancenreichen Sektoren (vgl. Tabelle 2) derzeit in Städten konzentriert sind, profitieren diese von den sich abzeichnenden Megatrend in besonderem Maße und weisen in der Regel hohe Sektorprognosewerte auf. Deutlich erkennbar sind die dunklen Einfärbungen sowohl in als auch rund um München, Frankfurt/Main, Berlin, Düsseldorf und Köln.

Die höchsten Werte unter allen 401 deutschen Landkreisen und kreisfreien Städten erreichen jedoch drei Städte im Osten Deutschlands — Jena, Gera (beide Thüringen) und Potsdam (Brandenburg). Jena verdankt seine Spitzenposition im Wesentlichen der hohen Beschäftigtenkonzentration im höchstbewerteten Trendsektor 72 „Forschung und Entwicklung". Gera ist die deutsche Hochburg im ebenfalls hoch gerankten Trendsektor 63 „Informationsdienstleistungen" mit einem Lokationsquotienten von 16,84. Potsdam ist breiter aufgestellt und punktet mit hohen Beschäftigtenanteilen in der Forschung und Entwicklung und dem weit oben gelisteten Trendbereich 82 „Erbringung von wirtschaftlichen Dienstleistungen". Stark ist Potsdam auch im Sektor 59 „Herstellung und Vertrieb von Filmen und Fernsehprogrammen, Tonstudios, Verlegen von Musik" (Sektortrendwert 7).

Weitere zehn im Osten Deutschlands gelegene Städte sind unter den Top 20 zu finden und weisen besonders gute Entwicklungsaussichten auf. Mit dabei sind die sächsischen Städte Leipzig, Chemnitz und Dresden, Schwerin aus Mecklenburg-Vorpommern, Frankfurt (Oder) und Cottbus aus Brandenburg, Halle an der Saale und Magdeburg aus Sachsen-Anhalt sowie das thüringische Erfurt und die Hauptstadt Berlin. Aus dem Westen sind Darmstadt (Hessen), Köln (Nordrhein-Westfalen), Heidelberg und Karlsruhe (beide Baden-Württemberg) sowie München (Bayern) dabei. Die beiden Landkreise, die ebenfalls eine Platzierung unter den ersten 20 erzielen, sind der bayrische Landkreis München (Platz 4) und der Main-Taunus-Kreis (Hessen), der von der räumlichen Nähe zur Stadt Frankfurt/Main profitiert (Platz 18). Mit Ausnahme von Darmstadt lagen in allen genannten 20 Regionen auch die Vergangenheitstrends über dem deutschlandweiten Durchschnitt, wobei insbesondere die Städte Potsdam, Frankfurt (Oder) und Jena weit überdurchschnittliche Werte erzielten.

### Stadt-Land-Ungleichheiten

Insgesamt kann festgestellt werden, dass der Strukturwandel im Zuge der Megatrends die Gefahr einer wirtschaftlichen Zweiteilung des Landes in städtische und ländliche Räume erhöht und bereits bestehende Ungleichheiten verstärken wird. Ab einer gewissen Größe sind verdichtete städtische Räume mit ihren Wirtschaftsstrukturen besonders gut an aktuelle Trends angepasst und für die Zukunft gerüstet, während ländliche und periphere Räume drohen, weiter ins Hintertreffen zu geraten. Ursächlich für diese Tendenz sind die guten Entwicklungsaussichten von Dienstleistungssektoren, insbesondere im Bereich der Informations- und Kommunikationstechnologien, die vor allem in Großstädten angesiedelt sind. Auf der anderen Seite stehen primäre Sektoren und einzelne Industrien, wie sie in ländlicheren Räumen zu finden sind, unter zunehmendem Veränderungsdruck. Richtet man den Blick ausschließlich auf die urbanen Räume, fällt auf, dass insbesondere ostdeutsche Großstädte heute über Wirtschaftsstrukturen verfügen, die eine gute Ausgangsposition für kommende Wandlungsprozesse bilden. Es ist zu erwarten, dass diese Städte wie bereits im vergangenen Jahrzehnt Beschäftigungsanteile in künftigen Trendsektoren weiter ausbauen können und sich im Zuge dessen bestehende Disparitäten zwischen ost- und westdeutschen Großstädten vermindern werden.

### Proaktiver Wandel benötigt Unterstützung durch Politik und öffentliche Hand

Es sollte nicht versucht werden, Veränderungen durch strukturkonservierende Politik zu verzögern. Ziel sollte es sein, den Wandel proaktiv zu gestalten. Damit Regionen die Chancen des Strukturwandels im Zuge der Megatrends nutzen können, sind jedoch unterstützende politische Entscheidungen, Handlungen und Vorleistungen notwendig (Grimm et al., 2021; Mönnig et al., 2019). Unerlässlich sind öffentliche Investitionen in Digitalisierung und die öffentliche Infrastruktur sowie in Bildung, Ausbildung und Forschung und Entwicklung. Auch Weiterbildung ist ein wesentlicher Faktor, wenn es darum geht, veränderten Anforderungsprofilen von Arbeitsplätzen begegnen und Arbeitslosigkeit vermeiden zu können. Notwendig ist zudem eine Innovationspolitik, die verstärkt auf Kooperation und Vernetzung abzielt, wobei Innovation als ganzheitlicher Prozess vieler unterschiedlicher Akteure zu begreifen ist (Pflüger, 2019; Horn, 2017).

**Title:** ***Megatrends and Structural Change – Which Regions Will Benefit?***
